# Healthcare organizations in crisis context: decision-making models and roles of CEOs

**DOI:** 10.1186/s12913-025-12420-6

**Published:** 2025-02-18

**Authors:** Anna Romiti, Mario Del Vecchio, Caterina Cavicchi, Emidia Vagnoni

**Affiliations:** 1 Department of Experimental and Clinical Medicine, University of Florence, 48 Viale Morgagni, 50134 Florence, Italy; 2 SDA Bocconi School of Management, 10 Via Sarfatti, Milan, 20136 Italy; 3 Department of Economics and Management, University of Ferrara, 11 Via Voltapaletto, 44121 Ferrara, Italy

**Keywords:** Crisis management, Healthcare, Decision-making process, Decision-making models

## Abstract

**Background:**

In times of crisis, decision-making can become a highly complex process that is shaped differently than in ordinary contexts. The literature presents different decision-making models that have not yet been thoroughly tested in practice. This paper aims to enhance our understanding of the distinctive characteristics of healthcare organizations’ decision-making models in crisis contexts, building upon and expanding the framework proposed by Arendt et al. (2005).

**Methods:**

Based on a qualitative research design, data were collected through semi-structured interviews with 49 CEOs of Italian healthcare organizations, including both local health authorities and independent hospitals.

**Results:**

CEOs’ decision-making processes demonstrated several commonalities: a shift from strategic issues to operational issues; a narrowed CEO focus on only a few areas of operations, and a significant concentration of the DMP in time and space. These shared elements provide insights into the nature of the centralization process adopted by CEOs in such situations. Additionally, we identified various decision-making models to manage the same crisis, with each model presenting a different level of involvement of other actors in the decision-making process, reflecting the CEO’s unique vision and background.

**Conclusions:**

This research makes a significant contribution to the growing literature on crisis management in three key ways. First, it emphasizes the importance of analyzing the decision-making process through the combined lens of different dimensions and phases. Second, it enhances the Arendt et al. (2015) model to include multiple critical dimensions of the decision-making process beyond the initial focus on decentralization, and demonstrates how, in a crisis context, the actors involved by the CEOs diverge from the original framework. Third, it offers new insights into the variables affecting the roles of the CEOs and other key actors in crisis-related decision-making, transcending the boundaries of role theory. In terms of managerial implications, reflecting on and interpreting the core of the organization’s decision-making processes, can support top management teams in navigating change, providing them a more robust and rational base for guiding the transition to a post-crisis “new normal”.

**Supplementary Information:**

The online version contains supplementary material available at 10.1186/s12913-025-12420-6.

## Introduction

Public health organizations in Italy have faced considerable pressure resulting from the COVID-19 pandemic, which has challenged many of the established models of governance and management [1]. In crisis management, how CEOs act and face unprecedented scenarios is a much-discussed topic [2, 3]. A stream of literature has been developed about decision-making models (DMMs) in crisis situations, drawing on different disciplines. Goldberg (2013) [4] attempted to develop a model to analyze decision-making inputs from a variety of perspectives, including emergency and business continuity professionals, to effectively predict the outcomes of decision-making and speed the return to normalcy after a crisis. Based on a case study, Vidaillet (2001) [5] concluded that decision-makers in a crisis context concentrate very little information and give greater weight to negative possibilities, and the feasibility of the options becomes a key point. In this regard, “formulating the problem, defining the alternatives and evaluating them are not separate sequential stages of the decision-making process” (p. 210). Dionne et al. (2018) [6] presented a multilevel theoretical model examining the interactive role that cognitions and emotions play in crisis decision-making, advancing the traditional crisis decision-making models of Sweeny (2008) [7] that applied to individuals experiencing crisis and of Lipshitz, Klein and Carroll (2006) [8] about the integration between naturalistic decision-making and organizational decision making. The literature on DMMs in crises has developed considering the roles of various inputs, stakeholders in complex organizations, communication, and emotions and cognition at both the individual and collective levels, while decision-making models (DMMs) that consider both the dimensions and steps of the decision-making process are still lacking.

Given the recent pandemic crisis, in the healthcare sector, particular emphasis has been placed on the role of CEOs in the decision-making process (DMP) [9–11]. The DMP at the strategic apex level has been described as both a sequence of steps or phases and as a set of different dimensions [12]. However, the abovementioned two perspectives have not been integrated into the literature. We use the consolidated DMM framework by Arendt et al. (2005) [13], which focuses on the steps of the DMP, integrated with the perspective of the DMP in terms of its dimensions [14], to analyze the DMP in the crisis context, considering both the sequence of steps (DMM) and the DMP dimensions. The framework by Arendt et al. (2005) [13] has been widely used in the literature to investigate the effect of the functional diversity of the TMT on firm performance by addressing the role of CEOs [15] or the influence of the TMT on firm strategy [16, 17]. Furthermore, the studies by Papadakis [14] have been largely used in strategic decision-making and human resource management research [18, 19]. Being well-recognized frameworks, we have used them to frame our theoretical lens.

Thus, this study’s central research objectives are as follows: How did the DMP, particularly the content and nature of CEOs’ decisions, change in healthcare organizations during the crisis (RQ1)? How are healthcare organizations’ DMMs shaped in times of crisis (RQ2)? How do the DMP dimensions characterize the different DMMs in healthcare organizations in times of crisis (RQ3)?

Our unit of analysis to study DMP is not “how to make a decision”, but who are the actors involved in DM by the CEO in the different stages of the DMP.

This study is meant to fill the literature gap related to DMMs that can be adopted in times of crisis since the importance of using the experience of previous crises to address new crises has been recognized [20].

This research uses a sample of Italian public healthcare organizations (HCOs); consistent with a qualitative approach, data about the decision-making process used by CEOs during the pandemic are gathered, and how the crisis affected the main dimensions of the DMP is analyzed. Therefore, the different DMMs emerging from the analysis are discussed in connection with the literature.

The literature on DMMs focuses on the level of hierarchical decentralization, which is an important dimension for characterizing DMPs [13]. From the theoretical perspective, we will extend our attention to the literature analyzing the other dimensions of the DMP (rationality/comprehensiveness, politicization and problem-solving dissension, lateral communication, formalization) [14, 21] to identify similarities and differences among DMMs in crisis situations. This study aims to enrich the model of Arendt et al. (2005) [13] by considering not only the hierarchical decentralization dimension, as in the Arendt et al. (2005) [13] framework, but also other important dimensions of DMPs [22]. Furthermore, the study, as far as the authors are aware, is one of the first to apply a DMM created for the private sector to examine the role of the CEOs of public organizations in decision-making during unprecedented crises, delving into the antecedents of role taking and role multiplicity [23]. Functional, relational, and structural antecedents help comprehend how the CEO managed the involvement of other organizational actors in the DMP in a crisis related context characterized by high environmental uncertainty.

From the managerial perspective, this study aims to highlight the characteristics of the decision-making process in a long-lasting crisis [24] to provide decision-makers with useful knowledge for making more effective decisions while also considering the risk that healthcare organizations will face an unforeseen crisis in the future [14, 25].

The remainder of this paper is organized as follows. Section "Background" analyzes the role of decision-makers in the strategic process and the peculiarities of decision-making during crises; Section "Methodology and methods" discusses the methodology; Section "Results" presents the results of the qualitative analysis; and finally, Section "Discussion" and "Conclusions" provide discussions and conclusions.

## Background

### The DMP at the top management level

Decision-making at the top management level is often defined as a set of “infrequent” decisions usually focused on the strategy ([26], p.17).

Theory on strategic decisions emphasizes different characteristics; Mintzberg et al. (1976) ([27], p. 246) defined a strategic decision as “one which is important in terms of the actions taken, the resources committed, or the precedents set”. Shivakumar (2014) [28] referred to strategic decisions by providing a taxonomy based on how much a decision is reversible (i.e., commitment) or influences the scope of the firm (i.e., strategic, neostrategic, tactical, and operational). A strategic decision significantly impacts both the firm’s commitment and its scope.

Decision-making at the top management level has also been studied with a dynamic approach in terms of DMP. In this vein, DMP has been described according to two major perspectives: as a sequence of steps or through the use of a set of different dimensions [12, 29]. Both perspectives complement each other and will be revised to analyze a complex phenomenon with blurred features.

Starting from considering DMP as a phenomenon that occurs on a set of different dimensions, Papadakis et al. (1998) [14], who conducted one of the most sophisticated studies in this field [21], suggested examining strategic decision-making processes (SDMPs) considering dimensions such as rationality/comprehensiveness, formalization, hierarchical decentralization, lateral communication, politicization and problem-solving dissension. This approach, among the most used approaches in the SDMP literature, will be better examined in paragraph “Dimensions of the DMP in crisis”.

### DMP as sequence of steps: the role of the decision-maker

In defining SDMP, some schools of thought, such as “environmental determinism”, emphasize the role of exogenous components, stating that strategic processes “are adaptations to external opportunities, threats, constraints, and other features of the environment. The role of managers is merely to facilitate this adaptation” ([30], p.371).

Other studies suggest that the definition of strategic processes should emphasize the endogenous component. The core theory is the “upper echelons” theory, which underlines the crucial role of the top management team (TMT) in the DMP [31, 32]. In this sense, the CEO and TMT are considered the main players influencing corporate strategies. Many studies focus on a specific dimension, particularly on the level of hierarchical decentralization [12], which in turn can be influenced by the TMT and CEO tenure. In particular, longer CEO tenure increases the level of involvement of middle management in decision-making [29].

By analyzing the phenomenon of hierarchical decentralization, other studies adopt a perspective based on the phases of the DMP, identifying the role of decision-makers in the sequence of steps that characterize the process. In particular, these studies examine the levels of involvement of the components of the strategic apex (CEO and TMT) and other “advisors” in the various phases of the DMP and, on these bases, propose different DMMs that are the result of several elements: the corporate environment, managerial vision and perceptions, and other internal/external forces [30].

Using different phases of the DMP (gathering information, processing and interpreting information, recommending and making decisions), Arendt et al. (2005) [13] identified three decision-making models: the CEO model, wherein the CEO adopts an autocratic style; the CEO–Adviser model (elitist DMM); and the TMT model (considered a democratic decision-making style). Given this framework for strategic decisions, CEOs involve different individuals of the organization and from different hierarchical levels. The result of these choices has also been called “strategic leadership constellations” [33], and the selection of the individuals who are involved in the constellations can depend on both the context and the environment [34].

In the CEO model, all strategic processes are governed by CEOs who make decisions based on their own ideas, with or without the support of information gathered by their colleagues [35]. In the CEO–Adviser model, different advisers are included in the gathering, processing, and interpreting sequence; the CEO considers the adviser’s recommendations but makes the decisions alone [13]. In the third model, the TMT model, the decision-maker is a collective body, the TMT, whose members gather, process, and interpret information and jointly develop and implement strategies with the CEO [13]. In this model, the choices are discussed in a team that makes the decisions [35].

The three models have not yet been tested in crisis, and the literature does not show how DMMs can change in crisis times. Healthcare organizations are complex and key to the development of a society, and their CEOs play a key role in strategic decision-making. In a crisis context, CEOs’ decision-making may affect both the setting of strategic goals and the natural function of the organization itself. Thus, this context can be studied using the framework of Arendt et al. (2005) [13]. Referring to Arendt et al.’s (2005) [13] models, the literature calls for studies that identify the antecedents of the involvement of individuals in the SDMP and focus on how CEOs manage this involvement in the different phases of the DMP [36]. Literature has shown how variables such as the CEOs experience and leadership style (i.e., functional antecedents), social interactions (i.e., relational antecedents) and power structure (i.e., structural antecedents), individually can affect key actors’ involvement in the DMP, especially in conditions of environmental uncertainty. Nevertheless, scant research has been dedicated to study how these antecedents, in an integrated manner, can affect role taking and role multiplicity in time of unprecedent crises [23, 37]. While role taking relates to how the CEO engage with other members organizing tasks, sharing knowledge and developing leadership, role multiplicity refers to how roles within a group intersect and influence interactions and behavioral outcomes [23, 38, 39]. Furthermore, given the complexity of the DMP and the variety of variables affecting the role of the CEO in the DMP, this paper focuses on DMMs, aiming to identify the dimensions that characterize DMMs in times of crisis given the characteristics of the DMP.

Therefore, the following research questions are proposed to deepen the understanding of the DMP and DMMs in the healthcare organizations, from the perspective of the CEOs:

- RQ1: How did the DMP, particularly the content and nature of CEOs’ decisions, change in healthcare organizations during the crisis? RQ2: How are healthcare organizations’ decision-making models (DMMs) shaped in times of crisis?

### Dimensions of the DMP in crises

The DMP has also been studied from the crisis management perspective. Organizational crisis is defined as an event considered “highly salient, unexpected, and potentially disruptive” [40]. Although each crisis has specific characteristics, several common traits have been recognized: high ambiguity, time pressure, and frequently changing conditions [41]. All these factors require rapid responses and can threaten organizational survival [10].

In the crisis management literature, decision-specific characteristics or contextual variables associated with the crisis (e.g., decision uncertainty and time pressure) influence the DMP dimensions. In particular, Papadakis et al. (1998) [14] analyzed the relationships among decision-specific characteristics and some DMP dimensions, such as rationality/comprehensiveness, politicization and problem-solving dissension, hierarchical decentralization, lateral communication, and formalization. Below, each of the DMP dimensions is briefly explained, and then the relationships are summarized.

The degree of *rationality/comprehensiveness* is linked to compliance with the “rational comprehensive” model that assumes a DMP based on well-identified variables and phases [42, 43]. Furthermore, when considering the cognitive side of DMPs, intuition remains a key factor in a DMP characterized by different approaches, including rational, intuitive, and improvisational [44].

In a politically based dimension (politicization), the SDMP can be considered a complex social process to which many stakeholders, inside and outside the organization, participate. From this perspective, the distribution of power among different stakeholders becomes relevant [45]. Indeed, decisions emerge as a result of a process in which decision-makers have different goals, forming alliances to achieve their goals and where the preferences of the most important group prevail [46]. This is particularly true in the public administration environment, where institutional dimensions assure the governance [47]. These concepts have been defined as the *level of politicization and problem-solving dissension* [14].

*Hierarchical decentralization* represents the degree of participation of different hierarchical layers in the phases of the SDMP. Low levels of hierarchical decentralization allow the decisions to be concentrated at the top of the organization. Conversely, high levels of hierarchical decentralization present more evenly distributed decision-making power [48].

Given a certain degree of hierarchical decentralization, *lateral communication* denotes the level of involvement in the SDMP of units at the same organizational level [49]; the CEO can receive advice from different points and levels of the hierarchy [36].

Decision-specific characteristics or contextual variables, such as a crisis, impact all the dimensions of the DMP described above.

The intensity of pressures generated by the crisis (i.e., time and other external pressure) decreases the level of lateral communication, as specifically perceived by workers in healthcare during the COVID-19 crisis [50], because during a crisis, the involvement of different organizational actors in the process becomes difficult [14].

Uncertainty about the environmental conditions, actions to be taken, and information to be considered increases the internal divergence of opinion. This requires more “bargaining”, increasing the level of *politicization and problem-solving dissent* [14], which could impair the effectiveness of the SDMP [46]. As confirmed in another study, during a crisis, decision-makers may face the competing interests of different stakeholders; in these participants, the use of power can be an important tool for solving a crisis [22].

Regarding the dimension *formalization*, the results are not consistent. Although it has been argued that uncertainty decreases the *use of formalized rules and procedures* in DMPs [51], recent studies have shown that during a crisis, formalization and delineating roles and responsibilities facilitate quicker decision-making through the creation of a crisis management team/unit [22].

In terms of the level of *rationality/comprehensiveness*, Huang and Pearce (2015) [52] argued that decision-making in crisis conditions is based on *intuition*. However, for intuition to operate as an effective answer, expertise based on years of *experience* and practice in decision-making is crucial [53]. Given the intuitive nature of decision-making in crises, Sayegh et al. (2004) [10] added that tacit knowledge and *emotional response* play important roles. Emotional response (e.g., empathy) is considered beneficial in terms of access to crisis-related information, the level of stakeholder appreciation, and the level of commitment to the organization’s relational system [54].

Moreover, improvisation is considered useful during crises, as managers are “forced” to improvise [55]. Referring to crises such as the COVID-19 pandemic, some authors have argued about the usefulness of *improvisation* [56]. Similarly, other authors [11] have enriched the debate on decision-making in crises and argued that comprehensive and intuitive decision-making can be combined to enable top management to make improvised decisions.

*The level of hierarchical decentralization* is often discussed in the field of the SDMP in crisis management. Centralization ensures a faster crisis response [9], without the need to establish a consensus in an environment characterized by high uncertainty [47], rapid changes [57], or the viability of drastic decisions [49]. In contrast, Boin and T’Art (2010) [58] suggested that the centralization process does not always improve the operational response capacity.

Regarding the specific crisis of the COVID-19 pandemic, some authors have proven that a top-down approach can challenge the adaptation of workers to new situations in the specific setting of healthcare [50]. In studies adopting more contingent perspectives, the level of centralization is linked to the crisis intensity or phase. Crises of mild intensity are frequently linked to decentralization; conversely, high-intensity crises are associated with authority centralization [14, 59]. Moreover, in the early stages of a crisis, centralization is an expected outcome. In later stages, the response can be decentralized [60].

Flexibility in terms of distributed leadership and a less hierarchical structure is considered an important lever for other authors to respond to a high degree of uncertainty surrounding a global crisis such as a pandemic [61]. In the same vein, Lloyd-Smith (2020) [56] argued that hierarchical decisions are inappropriate in a crisis because all relevant, specific knowledge and expertise are located at lower levels of the organization. Other authors show the importance of collaborative solutions between units of the same organization in terms of creating new expert groups or complementing existing ones [62]. In other words, an increase in *lateral communication* is considered useful in terms of pandemic governance.

In conclusion, on the one hand, we can observe studies that analyze various DMMs that are based on only one dimension of the SDMP (the level of hierarchical decentralization) [13], and on the other hand, there are no studies that show the other characteristics assumed by the SDMP (formalization, rationality/comprehensiveness, lateral communication, politicization and problem-solving dissension) in each DMM.

Finally, to the best of our knowledge, no studies have focused on how crises affect SDMP both in terms of their impact on different dimensions of the process and on DMMs [12]. Empirical studies analyzing the interactions between contextual variables and SDMP dimensions can help organizations design decisional processes in a crisis [63]. Therefore, this study focuses on the following research question:

- RQ3: How do the DMP dimensions characterize the different DMMs in healthcare organizations in times of crisis?

As already mentioned above, many factors influence the strategic decision-making process. In particular, among the most relevant are recognized the top management characteristics (CEO and TMT), decision-specific characteristics and contextual factors (external and internal) [14]. Considering the crucial role assigned to the top management and decision-specific characteristics on SDMP outcomes [], our paper focuses on the role of the CEO and on the decision process characteristics of DMP. In this perspective we use the “phase model” that focus on decisions taken by a focal actor [64], the CEOs, using the Arendt et al. (2005) model [13].

## Methodology and methods

### Research design

The aim of this paper was to gain an understanding of the decision-making processes in times of crisis by identifying their characteristics, how DMMs are shaped and how DMP dimensions characterize different DMMs.

Given the aim of the study to deepen the understanding of “how” the DMM and the DMP at the top level of the healthcare organizations are shaped by a crisis context, a qualitative research design was developed. Qualitative research is suitable to answer the research questions, as doing so entails gathering data related to the CEOs’ experience of the DMP during the crisis, more precisely, given the pandemic, how they made decisions, how they involved others, how they perceived their decision-making space, etc. This study applies a qualitative “individual-based, one-off” interview research design ([65], p.86), as this approach is particularly suited for revealing prominent experiences in terms of decision-making across the group of the healthcare organizations’ CEOs during the COVID-19 pandemic. This type of research design is well recognized in the literature and has informed a variety of scholars’ research, including McNulty and Pettigrew (1999) [66]; nevertheless, the main rationale behind the research design choice is that a large and diverse group of interviewees can better reveal what is general and what is particular among a sample of respondents as well as the differences among CEOs’ views and discourses in relation to the DMP [67, 68]. Based on the literature, an interview protocol was defined and tested through a pilot study to gather feedback on its comprehensiveness and understandability; then, the data collection from the CEOs was launched. Archival data about the protocols adopted to address the pandemic, the services provided to patients, and the regional health environment were also used.

### Study setting

The Italian National Healthcare Service (NHS) was chosen as the study setting as it is suitable for exploring the DMP in crises in a public sector context, particularly given that Italy was the first European country hit by the COVID-19 pandemic, which resulted in convenient access for the researchers. Furthermore, given that the NHS structure and organization are based on regional needs, the reaction to the crisis has allowed the deployment of different organizational features. In terms of the COVID-19 pandemic, the outbreak affected regions with different levels of severity and in different time frames. Thus, the Italian setting allows for the deepening of understanding for how DMPs have been shaped by the crisis and identifying the elements characterizing the different DMMs adopted by the CEOs of HCOs in heterogeneous regional settings, i.e., regional healthcare systems.

The Italian NHS is government funded, with 203 HCOs with juridical and managerial autonomy responsible for providing services. The NHS is a federalist system; the state defines the levels of services to be guaranteed across the country, while regions plan and manage healthcare service provision based on their specific policies and regulations. New Public Management (NPM) reforms of the Nineties led the actual organization of the Italian NHS. In each region, three different types of public organizations can be found. The *Aziende Sanitarie Locali* are local health organizations (LHOs) providing prevention, primary care, and acute care services. The *Aziende Ospedaliere* are independent hospitals (IHs) that gained juridical and managerial autonomy from LHOs, due to NPM reforms, and provide specialist outpatient services, acute care, and rehabilitation treatments. The *Istituti di Ricovero e Cura a Carattere Scientifico* are specialized hospitals (SHs) that focus on the research and care of specific diseases. Given the organizational autonomy of the regions, sometimes agencies in charge of emergency management (the regional health emergency agencies, RHEAs) can also be found.

LHOs, IHs, SHs are run by a general director (i.e., the CEO) appointed by the regional government. The CEO in turns, appoints (and is supported by) a Clinical Director and an Administrative Director. Together, they form a triad (the TMT) which is the formal governing authority of the organization [69]. Furthermore, the CEO is also supported by a management committee exercising a consulting function related to the governance of clinical activities. In SHs one can also found a steering council in charge of verifying the congruence of the hospital's activity with the regional objectives.

In LHOs, IHs, SHs, the TMT may cover part of the roles and functions that, in differently structured healthcare systems, are pertinent to a board’s responsibility [69]. Thus, as leaders of the HCOs, the CEOs were on the front line and were participating in decision-making during the crisis, which was key to the overall TMT and professionals.

### Data collection

As mentioned in Section "Research design", most of the data were collected first through semi-structured interviews that were conducted face-to-face from January to July 2021, when the pandemic was largely finished, and HCOs’ CEOs were approaching a “back to normality” period. The research team was composed of three senior researchers and one junior researcher.

All interviews were conducted by the same senior researcher following the interview protocol specifically developed for this study (Appendix 1). The other researchers all participated in the interviews remotely using Google Meet. All participants provided consent to participate in the interviews. Each interview lasted between 1.5 and 2 h, was recorded with the permission of the interviewees, and was transcribed verbatim. In this regard, reflective notes and researchers’ subsequent meetings helped bring out early reflections on the conducted interviews and improved the ability to identify the emerging topics to be deepened as the data collection progressed. Although qualitative interviews are common, they can lead to ‘taken-for-grantedness’ and a lack of critical attention to their use [70]. There are many aspects of qualitative interviews that might be taken for granted; thus, researchers’ ongoing commitment to reflection is important.

The second round of data collection was conducted through archival data related to the specific HCO whose CEO participated in the study and to the regional context to which the HCO belonged to in order to inform only the researchers on the characteristics of the regional context.

### Participants

Given the study setting, 49 CEOs of HCOs from both public local health authorities and public independent hospitals participated in the study. Researchers adopted a purposive sampling strategy based on a theoretical sampling technique to select CEOs [71]. This technique is based on selecting and adding participants during progressive data collection and analysis; we added participants to reach a maximum regional variation, even in terms of different crisis outbreaks in different regional health services, as confirmed by recent studies investigating the impact of the crisis in the Italian NHS [72].

Finally, HCOs’ CEOs from 12 different Italian regions were sampled: 21 from northern Italian regions, 17 from central Italian regions, and 11 from southern Italian regions. Table 1 shows the sociodemographic characteristics of the sample of interviewed participants.

**Table 1:** Sociodemographic characteristics of the sample

CEO	Age (2021)	Years in the organization in which she/he was CEO in the 2021	Years as CEO (until 2021)
1	65	7	12
2	63	2	6
3	52	2	5
4	52	3	6
5	52	2	2
6	69 (2020)	1 (2020)	21 (2020)
7	59	6	11
8	51	7	7
9	52	2	2
10	60	3	7
11	62	1	7
12	57	1	1
13	61	1	1
14	62	3	14
15	60	1	2
16	54	1	2
17	61	8	11
18	54	7	7
19	50	3	3
20	69 (2020)	10 (2020)	10 (2020)
21	52	6	6
22	59	3	5
23	63	3	6
24	59	2	3
25	61	16	16
26	61	6	6
27	60	1	6
28	53	2	8
29	55	9	9
30	65	5	17
31	64 (2020)	1 (2020)	13
32	59	3	6
33	63	3	3
34	57	2	6
35	60	3	3
36	65	2	13
37	58	8 (2020)	9
38	49	1	1
39	59	5	5
40	58	2	4
41	58	2	13
42	62	5	7
43	64	3	10
44	61	2	2
45	63	2	10
46	58	3	9
47	57	1	5
48	62	3	3
49	61	1	15
**Average**	**59**	**4**	**7**

### Data analysis

The data were analyzed via deductive content analysis, a method adopted to apply a previous theory in a new context [73]. Thus, the structure of the analysis is based on previous literature to help the researchers identify the main concepts in the text of the interviews.

The data analysis process was then organized following the steps below.

In the first step, to assign the variables to different DMMs [13], the involvement of different actors in the DMP in the different stages of the process was verified. For this purpose, starting from the interviews’ transcripts, texts were highlighted when the CEOs referred to the following: 1. the involvement of actors, 2. in which stage of the DMP they were involved and 3. if the involvement was meaningful. Then, the participants were categorized according to the different DMMs emerging from the literature; coding and systematizing the data through continuous comparisons helped identify the similarities and differences among the participants and to associate those similarities and differences with the DMMs. Senior and junior researchers independently coded the transcripts using the NVivo software manual coding function. Some differences in the coding were resolved through ongoing discussion to achieve consensus between researchers [74].

In the second step, for each interview’s transcription, the parts of text in which the CEOs referred to the relevant characteristics of the DMP’s dimensions, namely, rationality/comprehensiveness, politicization and problem-solving dissension, lateral communication and level of formalization, were highlighted. In more detail, given the interview transcripts:

- In terms of rationality/comprehensiveness, the prevalence of intuitive versus rational elements in the different steps of the DMP was highlighted. In this case, we verified whether the level of rationality was primarily based on expertise developed through years of experience and/or background;
- To identify the level of politicization and problem-solving dissension, we focused on the sentences in which the CEO reported difficulties or needed to manage any disagreements with other actors (regions or CEOs of other organizations) in the different steps of the DMP;
- To analyze the level of lateral communication, we focused on the references made by the CEO to the degree of involvement of horizontal levels in the different steps of the DMP. If the CEO makes decisions according to the organizational hierarchy in the DMP, the degree of involvement of horizontal levels or lateral communication was considered low. However, if the CEO based the DMP on involving key figures, independent of their hierarchical placement, the degree of involvement in lateral communication was considered high;
- In terms of formalization, the text in which the CEO referred to the use of formal versus informal rules and procedures was highlighted.

In the third step, for each interview, the parts of the text in which the CEOs referred to the involvement of the different stakeholders (manager, crisis unit, health director, administrative director, region, and stakeholders) in the different stages of the DMP were highlighted.

Finally, for each DMM, the prevalent characteristics of each dimension of the DMP mentioned above were identified. In this regard, the exemplary quotes were reported (in italics) to support the description of the results. The subcodes, codes and themes emerging from the coding process are shown in Table 2.

**Table 2:** Subcodes, codes and themes from the coding process

Themes	Codes	Sub codes
DMP dimensions	Rationality/comprehensiveness	The CEO relies heavily on its own experience when making decisions
		The CEO relies heavily on its own background when making decisions
	Politicization and problem-solving dissension	The CEO has established a positive relationship with the region
		The relationship between the CEO and the region has encountered some challenges
	Lateral communication	The CEO makes crisis-related decisions respecting the organizational hierarchy
		The CEO makes crisis-related decisions involving key figures, regardless of their hierarchical placement
	Level of formalization	The CEO frequently relies on formal procedures to make decisions
		The CEO frequently relies on informal procedures to make decisions
DMMs	CEO Model CEO-Adviser Model TMT Model	The CEO is the primary actor in all the stages of the DMP
	CEO-Adviser Model	The CEO actively engages multiple stakeholders in all the stages of the DMP
	TMT Model	The CEO actively engages the TMT in all the stages of the DMP

## Results

In the following subsections, we provide the study’s results. First, in the SubSection "Characteristics of the DMP in the crisis", we highlight how the content and nature of CEO decisions change and are common to all the CEOs interviewed (RQ1). Second, in SubSection "Different decision-making models during a crisis (level of hierarchical decentralization) and other characteristics of the DMP", we discuss the unique aspects of each DMM, identifying the parties involved at the various stages of the decision-making process and clarifying how each DMM is modified during a crisis compared to its original framework (RQ2). Finally, we examine how the DMP dimensions (rationality/comprehensiveness, politicization and problem-solving dissension, lateral communication and level of formalization) characterize each DMM (RQ3).

A preliminary observation is about a missing link between DMM and the characteristics of the single organization or groups of organizations. This cannot be considered as a result because it is, in large part, explained by the perspective of the study that looked, through the lens of DMMs, at how the CEO organizes the decisional process in a situation of intense pressure. If we had defined “the decision-making process” as the object of the study, in a different and more comprehensive manner, and considered “normal conditions”, we would probably have found some relations between the object and the organization.

### Characteristics of the DMP in the crisis

While gathering data, some elements have been found to be relatively invariant in characterizing the DMP in the crisis in all DMMs. At the same time, other dimensions of the DMP that presented differences among the DMMs were identified.

Regarding the common elements, the data collected allowed us to observe three characteristics: 1) a shift of the DMP from strategic issues toward operative issues; 2) a narrowed CEO’s focus on only a few areas of operations; and 3) a significant concentration of the DMP in time and space. These characteristics are described below in more depth.

#### Operationalizing processes: from strategy to operations

CEOs and the DMPs in which they are involved change the content and nature of their decisions. In a crisis, the most important decisions are those related to problems that need a practical response that addresses the needs arising from the crisis. This makes the DMP more operational in nature. Such a need to manage daily operations deprives organizations of the space for vision and decisions that produce long-term benefits. Essentially, CEOs’ decision-making spaces, which are typically oriented toward strategic decisions, become completely occupied by short-term operational decisions, normally taken care of by different parts of the organizational hierarchy: *This crisis has a temporal dimension that … has taken away the future of both people and organizations. This demanded an enormous level of concentration on contingency and everyday life.* (CEO 17).

#### Focusing decision-making on only specific areas of operation

CEOs need to focus on operations relevant to resolving specific problems that require clear and quick decisions in high-uncertainty contexts. Parts of the operations or services (e.g., ER, infectious disease departments) become strategic, and in that context, the organizations are evaluated based on the quality and speed of their response and results in such services: *Every morning, I did a quick briefing in the ER, Intensive Care Unit, and COVID-19 ward, so I had direct contact with the three most key units in the hospital. I had a constant relationship with these three units, ensuring my presence every day.* (CEO 36).

#### Compacting the DMP and reducing lateral communication

The COVID-19 pandemic imposed time pressures that shortened DMPs and concentrated them in specific “places” with only the actors that can provide useful information. Hence, the crisis decreased the need for *lateral communication*. Traditional collegial bodies become less effective and, in some organizations, are replaced with crisis units. From this perspective, CEOs typically interact with a small group of actors, including all stakeholders directly involved in the emergency, to expedite decision-making and implementation. Actors include professionals who hold information and/or clinical expertise related to the emergency: *I had to make a temporary COVID-19 task force for the emergency, with ten main participants, from hospital and territorial care, appointed to make decisions. I created roles for each area that, together with the Director in a committee, could make decisions more quickly.* (CEO 38).

The three common characteristics of the DMPs characterize a generalized contraction of the decisional space in terms of the actors involved and the processes and contents of the decisions.

By analyzing the other dimensions of the DMP, differences can be found in the following: *the degree* of *rationality/comprehensiveness, the level of politicization, the level of formalization,* and *the degree of hierarchical decentralization.*

### Rationality/comprehensiveness

During the pandemic, the speed required, and pressure produced by decisions led to a reduction in the time available to gather information and evaluate alternatives. This led to a reduction in the space for rational decision-making components in decisions. In this case, intuition played a more important role. However, differences could be found depending on the characteristics of the top management, as better explained in the section devoted to the different DMMs.

### Politicization and problem-solving dissension

In terms of politicization and problem-solving dissension, all CEOs underlined an increase in the need to manage disagreement among stakeholders, but the way in which critical issues were managed changed based on the type of organization.

### Formalization

Regarding this dimension, two types of formalization were identified: ex post and ex ante formalization. Ex-ante formalization refers the proceduralization or the predefinition of some steps of the DMP. Ex post formalization refers to accountability of the process in terms of providing documentary evidence of the effect of the DMP.

Regarding ex-ante formalization, we did not find homogeneity, while in ex-post formalization, there was a general tendency from all CEOs to structure at least the role of the advisor.

In other words, all CEOs reduced procedures on the one hand, but for some, the need to document and prepare formal accountability of processes arose.

### Hierarchical decentralization

We found three completely different answers from CEOs in terms of hierarchical decentralization. Given the relevance of the issue, these items will be analyzed more deeply in the next subsection.

### Different decision-making models during a crisis (level of hierarchical decentralization) and other characteristics of the DMP

**Fig. 1:**
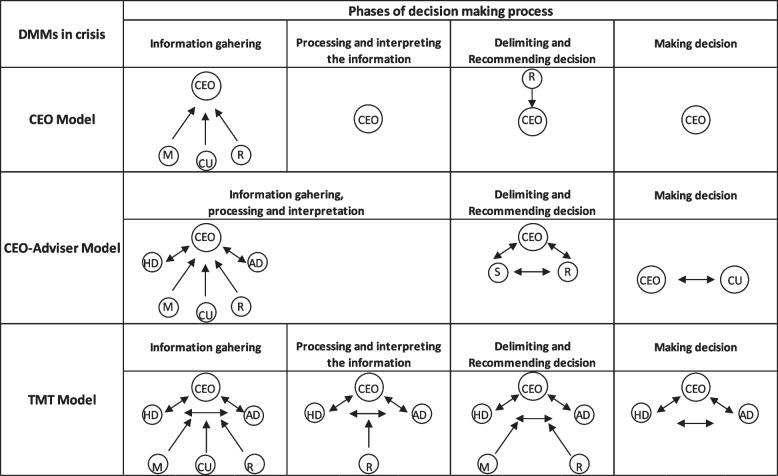
Models and phases of decision-making. M: manager (professionals responsible for organizational unit); CU: crisis unit; HD: health director; AD: administrative director; R: region; S: stakeholders. Source: Our adaptation by Arendt et al., 2005 [13]

The different DMMs were analyzed based on the framework proposed by Arendt et al. (2005) [8], which shows how different stakeholders participate in the different DMP stages (see Fig. 1). The framework was adapted, particularly in the third phase of the decision-making process, as follows: the “recommending decisions” stage was replaced with “delimiting and recommending decisions” stage, as all the organizations considered belonged to a regional group, and the region could intervene and modify the decisional space allowed for the CEO.

Based on the different levels of centralization, three clusters were identified and linked to the different models suggested by the framework: the *CEO model, the CEO-Adviser model, and the TMT model*. All types of HCOs and regions analyzed are represented within each group. The structural elements and sociodemographic characteristics of the CEOs in each model are shown in Table 3.

**Table 3:**
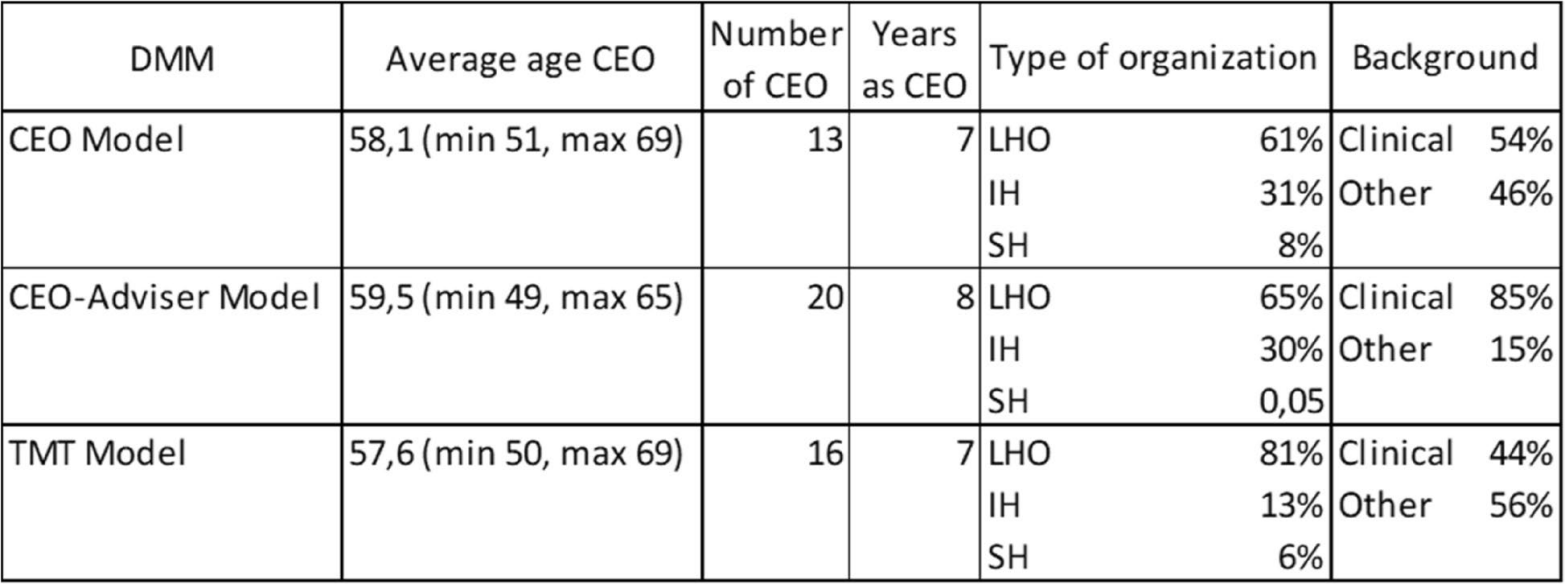
Sociodemographic characteristics and structural elements of each DMM

For each model, first, the dimensions that characterize the DMP and then the features of the phases of the DMP are presented.

#### CEO Model

This model includes all organizations whose CEOs concentrate most of the decisional burden on themselves. In their opinion, centralization is made possible by their specific competences, such as their technical background or previous experience. These are the subjective basis for a rational decisional process.

Several CEOs considered their technical background to be a legitimate basis for decision-making: *Having a clinical background with a specialization in healthcare emergencies, I had some advantage. I had a good cognitive framework … that in this case, made me go back to the basics, the essential things you cannot fail at, and, from that point, gradually build more refined solutions.* (CEO 2).

Others underlined their personal experience: *I had 20 years of experience as a director of hospitals. I have quite clear ideas about how to redesign and reorganize hospitals because I have done it many times.* (CEO 13).

In the context of increasing the degree of divergence of the actors' views, the CEOs of this cluster perceived the greater prominence of the region as an increase in the degree of politicization: *The region was involved in the decisions about the operations… but I have not limited myself to act as certifier of the correct fulfillment. It was not easy to combine my vision about what was happening in the specific territories of my organization with the regional strategic vision.* (CEO 11).

From the point of view of formalization, the majority of CEOs belonging to this model were concerned with producing accountability (ex post formalization): *From the beginning of the pandemic, we immediately began to decide on the paths that changed at the hospital level, and I said, "The pandemic passes, but the problems will remain, we have to leave a trace of everything we do and what we will do.* (CEO 7).

### Information gathering

In the information gathering phase, CEOs involved different actors, including line managers, crisis units, and regions. Crises change the scope and nature of the information required in DMPs. Management committees (composed of all Department Chiefs) that institutionally support CEOs in information gathering and decision-making are replaced by a crisis unit in which all relevant information sources are included.

Crisis unit composition is based on the ability of the subjects to respond to the operational needs dictated by the crisis regardless of their formal position: *We created a crisis unit wherein members of the Management Committee were not involved and the Board of Directors was completely bypassed. In the crisis unit, different people were involved according to their knowledge of critical organizational mechanisms.* (CEO 6).

### Processing and interpreting the information

CEOs chose to centralize the task of processing and interpreting the information on themselves, and only sometimes other members of the organization were included: *If you truly wanted to lead the organization, you had to centralize decisions. Everyone could offer their interpretation of the information, but at the end of the day, I take responsibility and make the decisions.* (CEO 2).

### Delimiting and recommending decisions

In the third phase of the DMP, delimiting and recommending decisions, CEOs perceived the region as an actor that limits the decisional space and the CEO’s role: *The Regional Crisis Unit centralized the distribution and use of beds, especially those in intensive care and subintensive care… The region mixes technical and political roles to give very specific provisions to the CEOs.* (CEO 12).

### Making decisions

At the core of the CEO model is the absolute role of the CEO in the decisional phase. Management committees, which normally share the responsibility of decisions with CEOs, were bypassed and were only just informed of the decisions to maintain the speed needed: *The decisions I made were mainly made by me going on the field … I called the head of the structures and explained to him that it must be done.* (CEO 5).

#### CEO-Adviser model

The CEOs of this cluster are characterized by collaborative relationships with both the members of the organization and other external stakeholders. From the CEO’s perspective, this is considered an aspect that plays an important role in facilitating crisis management and at the same time reducing the problems linked to the management of the political dimension (politicization): *The management of the pandemic in the territory of this organization depends on what can be done in agreement with the local institutional coordination.* (CEO 38).

The collaborative relationship allows CEOs to also create the basis of a decision-making mode that prefers rationality to intuition. The CEOs were aware that building a deeper knowledge of the phenomenon to make a better decision can still be a more tiring process: *When you decide it is better not to make mistakes, to have all the possible information… you have to create a group… if you involve and make more effort you will have it much easier in the subsequent management.* (CEO 40).

Regarding the formalization dimension, no homogeneous behavior of the CEOs in this cluster was observed. There were CEOs who preferred the formalization of DMPs and others who also made frequent use of nonformalized decisional processes.

In this cluster, the first two stages of the DMP (information gathering and processing and interpreting the information) cannot be separated because they occur simultaneously.

### Information gathering, processing and interpretation

In this model, all the main internal individual and collective actors participated in the *information gathering, processing and interpretation*: managers, crisis units, regions, and TMTs. A CEO states: *The health director, the administrative director, and I, with other people, used to have our chat on WhatsApp, where we shared every bit of information we came across… so we gradually gathered pieces of information from other contexts to help us to understand what we would have to face.* (CEO 31).

### Delimiting and recommending decisions

CEOs in this group perceived great autonomy and responsibility in their role. Although the region had a critical role in the choices of single organizations and narrowed the CEO’s decisional space, this was perceived not as a limitation but as a support in a difficult exercise of autonomy. Discussing and sharing choices at the regional level allowed the organizations to build a common managerial and technical responses throughout the territory and made CEOs feel thoroughly involved: *With the region, we have grown a lot because, with the region, we have also defined the model response to the crisis. The way in which we have interacted with the region and how the region led us resulted in a unified and homogeneous model. There is a shared approach, and the framework is the same for all organizations, being aware that each of us can adapt it to the different necessities of his context.* (CEO 42).

### Making decisions

CEOs primarily needed to involve the crisis unit in making decisions. The crisis unit was not only a staff unit but also a collegial body. On some occasions, their decisions were delegated to that unit that could have different compositions according to the decisions to be made: *Decisions were discussed and then made in the crisis units … A crisis unit with variable geometry depending on the object to be treated in the different phases of the crises was implemented”.* (CEO 34).

#### TMT model

In this cluster, the TMTs (CEOs, health directors, administrative directors, and, in some cases, health and social directors) played a critical role in all stages of the DMP during the crisis.

The element that the CEOs of this cluster shared is an empathic relationship with professionals that helped the development of a rational DMP: *The experience of COVID-19 has really consolidated the relationship with operators. I work with everyone, and, with the people I work with, there is a relationship that really goes beyond work. This is positive, and this also facilitates the job.* (CEO 14) They underlined the crucial role of collaboration with stakeholders to mitigate the problems that may arise from politicization: *The system worked well because I have taken care of the whole system of institutional and organizational relationships. In that period, I learned how powerful relationships are. If relationships work well, everything becomes much easier; in fact, now I’m investing much more time and effort in relationships and communication.* (CEO 18).

Most CEOs in this model preferred a formalized approach that included the formalization of different roles in the DMP due to the formalization of the institutional role of the TMT. Recoding procedures, they preferred an ex-ante formalization that reduced the problem of accountability (ex-post formalization): *The decisions were made in the crisis unit that we set up immediately and I wanted to formalize that.* (CEO 22).

### Information gathering

All members of the TMT participated at this first stage of the DMP. In some organizations, the TMT is composed of a fourth member—the health and social director—that collaborated with other members of the TMT to collect information: *In the information gathering, an important contribution was made by my health director, my administrative director, and my health and social director.* (CEO 26).

An important role in information gathering was also played by the region, the managers and the crisis unit (CU)*.*

### Processing and interpreting information

According to this model, TMTs and the region were critical in processing and interpreting information: *From the first week of March, we have started—obviously on the initiative of the Regional Councilor—a daily call with all the CEOs of the region that has continued since then. Together, we formulated strategies and specific responses, sharing all the information, data, critical issues, and solutions among us and with the region.* (CEO 29).

### Delimiting and recommending decisions

The region also played an important role in delimiting and recommending some decisions: *It is true that from the epidemiological perspective, we did not have much information available on the situation, namely, on the spread of the epidemic. However, we had all the regional indications laying down the steps to be followed to reorganize the services.* (CEO 27).

Regional recommendations were discussed by the TMT, which, in turn, collected other information from all available sources and proceeded to identify the best solutions. Although the decisions were made by the strategic apex, information was also collected from professionals in the field who had direct knowledge and who provided support in the decisions*: The main decision was always made by me, with the Health Director, and sometimes with the administrative director, but we involved the anesthesiologist in charge of the two main hospitals because he is ultimately responsible for all the actions that follow our decisions.* (CEO 24).

### Make decisions

Decisions within this cluster were made predominantly within the TMT: *It was the strategic direction that made all the decisions and communicated them inside the organization. Decisions were made based on the information that emerged from the contacts with different employees, head of specialties, and department directors. We discussed and explained the reason why certain decisions were made.* (CEO 15).

The models and phases of decision-making are shown in Fig. 1.

## Discussion

The study revealed that the crisis changed some relevant dimensions of ordinary DMPs at the top management level. In this vein our study confirms the importance to study separately the decision making of CEO, from that of the other members of the TMT, for its “decisive role” in the organization [29]^.^

In response to RQ1, first, this study allows us to argue that both the content and nature of CEOs’ decisions changed, becoming more operational; although CEOs’ decision-making is usually dedicated to strategic decisions [26, 31], during a crisis, it focused on short-term operational decisions. Thus, the CEO had an unusual level of hierarchy in the DMP based on emerging need. With reference to this first result, our study confirms previous studies that underline the importance for a CEO to work alongside those who are holders of the available information because they are operationally located where the crisis is run [5]. Regarding the same previous studies, our study confirms that also in crises of significant magnitude, such as the one we analyzed, the attention of the CEOs must be more selective and focused.

Second, the focus of decision-making moved from considering the whole organization [28] to specific areas of operations, and third, CEOs concentrated the DMP, reducing lateral communication [36]. These characteristics of the DMP in crisis change the individuals that the CEOs needed to be involved in the DMP, creating different “strategic leadership constellations” [33]. These parameters change on the basis of DMMs. Thus, the study of strategic leadership constellations [33] is important because it allows us to better understand decision processes and outcomes [17] in a crisis.

In response to RQ2, however, different choices were found. The three DMMs of the framework used [13] withstood the test of the crisis. We found no convergence toward a single model.

The CEOs showed different responses in terms of the level of hierarchical decentralization in the crisis. The literature shows that this level depends on the type and severity of the crisis [14]. However, this study demonstrates that in the same crisis, different responses can be found, and these can be linked to different leadership styles (attitudes toward relationships and collegiality or the importance of the CEO assigned to this element for managing organizations) or to characteristics of the CEO, such as their background and experience (in terms of previous crisis management) and contextual characteristics, such as the perceived autonomy from the regional government [23].

In all of the observed DMMs, the stage of the DMP, “recommending the decision”, changes to “delimiting and recommending decisions” to include the role of the region in the DMP. This shows the specific nature of the relationships of the healthcare system in terms of the decisional space the region assigns to the CEOs, reducing the decisional space during the crisis to manage and support the emergency.

Considering the different DMMs, some differences from the model proposed by Arendt et al. (2005) [13] can be highlighted.

In the CEO model, during the crisis, the region and crisis unit joined the managers to support the CEOs in the gathering information phase. As a result, the centralized model (CEO model) reduced CEOs’ level of centralization, especially in terms of gathering information, as the CEO could not handle it alone and involved different actors in this step of the DMP.

According to the CEO–Adviser model, a widening of the actors involved by the CEO in the DMP leads to a lack of clarity in the separation of roles. The same actors play different roles, and it is no longer possible to distinguish the roles of the different actors in the different stages of the process. As a result, role multiplicity through boundary spanning, took place [23].

In part, the TMT model shares with the previous model the expansion of the actors as the DMP becomes more collegial, especially in the gathering of information, a step in which all the actors are involved to build consensus among them. Furthermore, as an institutional-based model, the relation with the region also becomes important.

These findings contribute to the literature on the effects of decision-makers on the DMP [46] and, in greater depth, attempt to provide an answer to the issue of how CEOs manage involvement in the different phases of the DMP [46] in a crisis context. Within each DMM, there is an adaptation to the original framework that could be useful even in ordinary times.

In response to RQ3, starting from the *rationality/comprehensiveness* dimension, this study allows us to argue that different cognitive approaches are considered useful during the crisis, and these can be integrated. If the intuition leads to different choices in the DMP [52], improvisation is thus considered useful in the same process [56]. However, previous background and experience, as functional features of the CEO’s role, considered useful in some studies [20, 52] were found to play important roles, particularly in the CEO model, in managing the crisis and pursuing a strategic vision, eventually overcoming any possible issue arising from power distribution (i.e. the CEO's relationship with the regional government and with the internal management committee). This result advance previous studies that called to verify the influence of past experience and behavior on the process of responding to a crisis [7]. The emotional and informative support of workers and stakeholders compensate for CEOs’ lack of experience and background in crisis management in deciding on the other two models. In the case of the CEO-Adviser model, collaboration with stakeholders is considered an element that can help in decision-making in the belief that cooperation with them enables better decisions, prioritizing the functionality of roles. Different actors and roles are then involved in decision-making to address operational issues that emerge from time to time in the crisis context. This confirms also that emotional response and empathy characterizing social interactions between the CEO and other key actors [54] can be considered the best lever to mobilize different competences to make decisions in some DMPs. In the case of the TMT model, the principal stakeholders that support the DMP are considered the TMT members. Our results advance the theory in individual and collective decisions during crisis [6] demonstrating that decision makers in crisis do not follow similar paths but they can show different decision-making models based on CEO choices, experience and background. They also confirm the crucial role of CEO’s experience in information exchange and integration not only with other TMT members, as previous research show, to cope with the crisis [15], but also with other stakeholders; this changes based on the different DMM chosen by the CEO.

The findings about the politicization dimension did not confirm previous results [14]. This dimension has high variability depending on the constellations [17, 33] that the CEO forms; sometimes, politicization increases during the crisis, while in others, the level of politicization is lower because of the collaboration that CEOs created with all the stakeholders. As a healthcare organization is part of a regional group, the region is present everywhere; however, there are differences in regional styles and perceptions among CEOs about decision-making autonomy.

In terms of rule formalization, this study shows different results with respect to previous studies where in emergency situations, managers always decrease the use of formal procedures and rules [51]. The public nature of the organizations analyzed may influence this outcome.

## Conclusions

Top management plays a central role in the life of every organization, especially when an organization struggles to survive a crisis. In this case, the relevant literature underlines how the CEO becomes the focal point for every decision under the close observation of all the personnel who look to the top for direction and reassurance. This sort of “inward” movement has very often been described as centralization [9], meaning that top management plays a wider and more important role in DMPs during crises than in ordinary times. The experience of Italian public HCOs during the long-lasting COVID-19 crisis has provided an opportunity to better understand such a change.

First, new DMPs and new roles for the different actors involved are, in part, the result of an objective and subjective shrinking of the decisional space available for CEOs’ decisions, in terms of areas that require the CEOs’ attention. Objectively, closing many services because of the pandemic as well as the importance given from the context to some parts of the organization (e.g., ER, infectious disease departments) against others should be considered. At the same time, subjectively, the CEO decides to exclude large units from his or her attention. From this perspective, “a smaller” (objective and subjective) organization makes it possible for the CEO to occupy a larger part of DMPs, including decisions directly linked to the operational sphere. In this case, a more appropriate definition would be “downward expansion and saturation of a limited space” rather than centralization. Therefore, the first contribution of this paper is to elucidate how the CEO's role evolves during a crisis, particularly highlighting the implications for its decision-making space [23]. This study provides a more nuanced explanation of this process, which the literature has broadly termed centralization. While the literature recognizes that centralization enables a swifter crisis response [52], the detailed characteristics of the centralization process have not been thoroughly explained until now, as our study attempted.

Second, how a given reconfigured decisional space is saturated is addressed, that is, which DMM is adopted. The sudden outbreak imposed a dramatic change in the DMP in search of decisional speed and operative effectiveness. Even if the study did not specifically focus on the determinants that led to the adoption of a specific DMM, the role of the CEO in shaping a new way of making decisions and governing cannot be underestimated. Under the pressure of the crisis, older roles and mechanisms were quickly abandoned, and every CEO chose how to rearrange the entire decision-making process. In this respect, it can be safely assumed that the model adopted during the crisis reflects, more clearly than the ordinary model, the inner vision and background of the single CEO.

From this perspective, the results of our study complement previous research showing that the selection of the individuals involved in the DMP depends on the context and the environment [34], shaping new “strategic leadership constellations” [33] in response to the needs of the crisis. Additionally, our study highlights that beyond the context, the CEO’s vision and background significantly influence the chosen DMM, and consequently, the role that they and the other actors play in the DMM.

In the aftermath of the pandemic, after two years of crisis, HCOs are gradually realigning their operations to a new normality, partially restoring the old mechanisms, in part incorporating the experiences of the last two years in their managerial designs and practices. In terms of managerial implications, a reflection and an interpretation of what has happened at the core of the organizational decisional processes may help TMTs in the present context of change, offering them a stronger and more rational base for guiding the transition to the new normality. Two elements, among others, should be carefully considered by the top management of HCOs. The first is the possibility of subjectively defining the decisional space, that is, focusing attention on the areas of overall operations that are key for achieving the institutional goals in each environment. The advantages of selectivity are a great lesson offered by the pandemic. The second is the effectiveness of decisions. The implementation phase has often been a weak point in public sector organizations where decision-making processes are strictly regulated, and sometimes, making decisions seems to be more important than the effect they generate. The necessity to tightly govern the implementation and translation of a decisional process in action has been another important lesson from the crisis.

In summary, from an academic point of view, this study contributes to the debate on DMPs in crisis situations and the role that the CEO can play, confirming the importance of examining them through the lens of different dimensions [14] and phases [13]. In other words, our study contributes to the literature on DMPs in three keyways. First, we propose integrating two approaches to the DMP at the strategic apex level, which are typically analyzed separately in the literature: one that describes DMP as a sequence of steps or phases [13] and another that views it as a set of different dimensions [14]. Second, this study applies these two approaches to a crisis context to examine how they can be adapted and integrated. From the first perspective, our results show how, in a crisis context, the actors involved in the decision-making process change with respect to each of Arendt et al.’s DMMs [13]. From the second perspective, the study demonstrated how the dimensions of the DMP [14] change according to the chosen DMM. In this way, the study enriches the model of Arendt et al. (2005) [13] by integrating the hierarchical decentralization dimension, the only dimension considered in the model [13], with other important dimensions of the DMP [22].

In this way, it is possible, on the one hand, to appreciate the effect of the crisis on each different dimension with reference to previous studies and, on the other hand, to cluster the variety of possible responses in a few models that can help the deepening of scholars’ understanding.

Third, the paper shed light on the variables affecting the roles of the CEOs and other key actors in a crisis-related decision-making context, cutting the boundaries of role theory specification and focusing on the different phases of the DMP characterizing the DMMs [23]. Thus, role theory, integrating functional, relational and structural variables, combined with the DMM theory, can inform future research on crisis management in public sector organizations.

To our knowledge, this is the first study that examines different CEOs’ DMMs in crisis and that, at the same time, explains the characteristics of the main dimensions of the DMP in each of these models. Future research could refer to this study to assess how the experiences and lessons from the COVID-19 pandemic would have affected the evolution of the DMP and the role of CEOs in the return to ordinary conditions.

The limitations of this study stem from the methodology chosen for the analysis, as it relies solely on CEOs’ perceptions of the decision-making process within a single crisis context. To enhance the findings, future research should consider the perspective of other internal and external stakeholders involved in various phases of the decision-making process. Additionally, examining multiple crisis contexts would help to confirm or challenge the results of this research.

## Supplementary Information

Supplementary Material 1.

## Data Availability

The data used in this manuscript are from recorded and transcribed interviews. It is not possible to share the research data publicly, since individual privacy could be compromised. The datasets are available from the corresponding author on reasonable request and with permission from interviewees.

## Abbreviations

DMP: Decision-making process
DMM: Decision-making model
M: Manager
CU: Crisis Unit
HD: Health director
AD: Administrative director
R: Region
S: Stakeholders
T: Top management team
