# Supplementary material for: Healthcare organizations in crisis context: decision-making models and roles of CEOs
Source: BMC Health Serv Res. 2025 Feb 18;25:273. doi: 10.1186/s12913-025-12420-6 (PMC11837454; doi:10.1186/s12913-025-12420-6)
Supplement: Supplementary file 1 — Supplementary Material 1. [file 12913_2025_12420_MOESM1_ESM.docx]

**Appendix 1**

**INTERVIEW GUIDE**

**Following a semi-structured interview guide, the main questions were:**

- What is your educational and professional background? And how long have you held the position of general director in the healthcare organisation under study?
- What are the main characteristics of the healthcare organisation you manage (for instance: catchment area, relevant healthcare facilities managed within the local territory, specialization in terms of health care services provided, etc.)
- How can you describe your experience as CEO of the HCO during Covid-19 period? In general, how has your role changed during that period?
- What were the main challenges you faced during the pandemic and what have been the main decisions or kind of decisions you have taken in response to these challenges?
- Would you describe the relation with the regional authority when it comes to the decision-making? Did the region affect your decision-making?
- Would you tell us about how you made decisions, and the extent to which you involved other bodies?
- How were crisis information for decision-making gathered and elaborated?
- Do you think that the decision-making process changed across the different phases of the pandemic? Eventually, how did it change?
- To what extent the decision-making process involved (and was affected by) the stakeholders?
